# Quality Properties of Innovative Goat Milk Kefir Enriched with Date Paste (*Phoenix dactylifera* L.) and Whey Derived from Goat Cheese Production

**DOI:** 10.3390/foods14101655

**Published:** 2025-05-08

**Authors:** Clara Muñoz-Bas, Nuria Muñoz-Tebar, Manuel Viuda-Martos, Raquel Lucas-González, José Ángel Pérez-Álvarez, Juana Fernández-López

**Affiliations:** IPOA Research Group, Institute for Agri-Food and Agri-Environmental Research and Innovation, Miguel Hernández University (CIAGRO-UMH), 03312 Orihuela, Spain; clara.munozb@umh.es (C.M.-B.); nmunoz@umh.es (N.M.-T.); mviuda@umh.es (M.V.-M.); raquel.lucasg@umh.es (R.L.-G.); ja.perez@umh.es (J.Á.P.-Á.)

**Keywords:** kefir, goat, date, coproducts, fortification

## Abstract

The aim of this work was to evaluate the impact of fortifying goat milk kefir with high-value ingredients (3% and 6% date paste, and 25% and 50% goat milk substitution with date–cheese whey), derived from the valorization of date coproducts, on its nutritional (proximate composition and mineral profile), technological (pH, acidity, viscosity, color, sugar and organic acid content), microbiological and sensory properties. Both ingredients enhanced the growth and stability of the kefir starter culture, thereby improving the probiotic potential of date-added kefir and also its nutritious quality (lower fat content and higher protein content). The mineral profile of kefir was improved only when the date paste was added. Date paste could be used as an ingredient in fortified kefir (up to 6%) without altering its flow properties because it was perfectly integrated within the milk matrix. The use of date–cheese whey as a goat milk substitution (>25%) decreased the typical kefir viscosity, inducing an excessive phase separation negatively valued by consumers. Consumers preferred the kefir with 6% date paste mainly due to its higher scores for aroma, flavor, sweetness and acidity.

## 1. Introduction

Dairy products are common and essential foods in the human diet due to the unique properties and components of milk (i.e., calcium content, protein and vitamins D and B12, among others) [1,2]. These nutrients are crucial for maintaining strong bones, supporting muscle health and promoting overall well-being [3,4]. Moreover, dairy can be a source of probiotics, which contribute to gut health and enhance digestion [4]. Additionally, the versatility of milk has enabled the production of a wide range of milk-based foods (fresh, aged, fermented, flavored, etc.) tailored to meet cultural preferences, regional availability and consumer demands. The dairy industry has leveraged this versatility to lead the way in developing new dairy products by the addition of health-promoting ingredients such as vitamins, dietary fiber, omega-3 fatty acids or tocopherols. This has resulted in functional and/or fortified dairy products that are highly accepted by current consumers [5,6,7].

In this sense, kefir is a self-carbonated alcoholic fermented milk, originating from the Caucasian Mountains, that can be made from any type of milk (cow, goat or sheep milk) [8,9]. The selection of goat milk versus cow milk for kefir production is mainly based on the better nutritional profile, digestibility and technological suitability [10,11]. Goat milk has higher levels of calcium, potassium and vitamin A compared to cow milk. Its fat globules are smaller and more easily digestible, making it a suitable alternative for individuals with lactose intolerance or a cow milk protein allergy [12]. The presence of medium-chain fatty acids in goat milk contributes to its potential health benefits, such as improved metabolism and enhanced immune function [10]. Additionally, goat milk exhibits excellent technological aptitude while maintaining its nutritional integrity and flavor profile.

The fermentation process of kefir involves the symbiotic growth of various microorganisms, including lactic acid bacteria (*Lactobacillus* spp., *Streptococcus* spp., *Lactococcus* spp. and *Leuconostoc* spp.), yeasts (*Saccharomyces* spp., *Candida* spp., *Torula* spp. and *Kluyveromyces* spp.) and acetic bacteria, which can help to restore the gut microbiota balance, thereby enhancing digestive health, immune function and overall well-being [13]. In the large intestine, probiotic bacteria play a key role in metabolizing dietary carbohydrates that would otherwise be indigestible (e.g., prebiotics), having a favorable influence on the metabolism of proteins and ammonia in the colon [14]. Likewise, lactic acid bacteria help absorb lactose and have a beneficial effect on many digestive system diseases [4].

Kefir is a refreshing fermented dairy product known for its smooth, flowing consistency, uniform and bright appearance and mild, yeast-like taste and flavor [15]. Additionally, it is a rich source of sugars, organic acids, alcohols and esters which are produced during fermentation [16]. Most kefir consumers choose it for its health benefits, while the main reason for non-consumption is a dislike of its taste [17]. Beyond probiotic microorganisms, there is a growing consumer interest in diets enriched with fruits and vegetables to improve both the functional and sensory qualities of food [18,19]. Also, consumers are increasingly concerned about the sustainability of the food production chain [20,21]. In this context, recent efforts have been made to valorize the coproducts from the agri-food industry, given their richness in valuable compounds such as dietary fiber, vitamins, sugars and polyphenolic compounds. This approach helps reduce waste production while contributing to the UN’s Sustainable Development Goal 12: Responsible Consumption and Production [22,23,24].

In this regard, coproducts from the production and commercialization of fresh dates (*Phoenix dactylifera* L.) have been processed by applying non-pollutant basic operations (e.g., grinding, soaking and drying) to obtain intermediate and stable high-value ingredients which are ready to be reinstated in the food chain [25]. The resulting ingredients (date water, date paste or date powder), which vary in appearance, physicochemical properties and composition, offer significant versatility for their incorporation in a wide range of food matrices [11,22,25]. Notably, these ingredients retain much of the nutritional value of the original raw material (fresh dates of the Confitera cv), being rich in sugars, organic acids, dietary fiber, minerals, vitamins and polyphenolic compounds [25,26,27]. In addition, some of them have demonstrated relevant biological properties such as a prebiotic effect [28], which is particularly significant in fermented dairy products [11,29,30].

Growing awareness on digestive health among consumers has forced manufacturers to develop new probiotic beverages. In Europe, sales of kefir are increasing because probiotic foods and drinks are popular. The global kefir market size was valued at USD 1.23 billion in 2019 and is projected to reach USD 2.40 billion by 2032. New product developments focusing on emerging trends such as organic, lactose-free, flavored beverages and with sustainable ingredients and process are expected to drive the growth of the kefir industry in the coming years. This last aspect (sustainability) is where our work is mainly focused, because the valorization of coproducts from the agri-food industry would contribute to reaching zero waste and to the circular economy. In any case, this fact will not be negative for the industry or for consumers.

The aim of this work was to evaluate the impact of fortifying goat milk kefir with high-value ingredients derived from date coproducts on its nutritional composition and physicochemical, microbiological and sensory properties.

## 2. Materials and Methods

### 2.1. Materials

The goat milk was collected from the farm of the Miguel Hernández University (Orihuela, Alicante, Spain) during the spring of 2024 and was kept refrigerated (4 °C) until its use less than 12 h later (protein: 3.2 g/100 g, fat: 4.6 g/100 g and carbohydrates: 4.3 g/100 g). Date paste (protein: 1.2 g/100 g, fat: 0.4 g/100 g and carbohydrates: 49.7 g/100 g) was obtained, as an intermediate ingredient, from the coproducts from fresh dates (Confitera cv.) from Elche (Alicante, Spain), following the process described by Muñoz-Bas et al. [25]. The whey (protein: 0.5 g/100 g, fat: 0.3 g/100 g and carbohydrates: 4.1 g/100 g) was obtained from the previous production of fresh goat’s cheese with 8% added date paste [11]. The starter culture (Kefir Yogotherm) was purchased from Abiasa (Pontevedra, Spain) and contained the following strains: *Lactococcus lactis* subsp. *lactis*, *Lactococcus lactis* subsp. *cremoris*, *Lactococcus lactis* subps. *diacetylactis*, *Lactobacillus rhamnosus* and *Kluyveromyces marxianus*.

### 2.2. Kefir Making

Goat milk was pasteurized at 60 °C for 30 min in a Thermomix TM6 Vorwerk (Wuppertal, Germany) and then cooled at 25 °C. Five batches of one liter each were made: one control kefir (C), two batches with 3% and 6% date paste (DP3 and DP6) and another two batches with a 25% and 50% replacement of milk by cheese whey (WH25 and WH50). First, the milk was mixed with the date paste or cheese whey and then the kefir starter culture was added at the dosage established by the company (1 g/L of milk). Subsequently, the kefir was poured into 100 mL containers and incubated for 20–22 h at 25 °C. Finally, the samples were refrigerated overnight (less than 24 h) until further analysis.

### 2.3. Kefir Analysis

#### 2.3.1. Proximate Composition

Moisture (AOAC 925.45) and ash (AOAC 923.03) were analyzed following AOAC methods [31]. On the other hand, fat, protein and totals solids were determined with a MilkoScan FT120 (FOSS, Hilleroed, Denmark) calibrated for cream. All measurements were performed in triplicate.

#### 2.3.2. Physicochemical Properties

##### pH and Acidity

The pH of the kefir was measured with a pH-meter Sension + pH31 (HACH Iberia, L’Hospitalet de Llobregat, Barcelona, Spain). On the other hand, acidity was measured by titration with NaOH 0.11 N using phenolphthalein as an indicator, with the results expressed as ^0^Dornic (^0^D). Acidity and pH were determined in triplicate.

##### Color Properties

CIELAB color coordinates (L*, a* and b*) were performed in triplicate and were measured using a spectrophotocolorimeter (Konica Minolta, Osaka, Japan) with a D65 illuminant and a 10° observer angle. From the color coordinates, the psychophysical attributes, chroma (C*), hue (H*) and the whiteness index (WI) [32] were calculated:

C* = (a*^2^ + b*^2^)^1/2^ (1)

H* = arctg b*/a*(2)

WI = 100 − [(100 − L*)^2^ + a*^2^ + b*^2^]^1/2^ (3)

##### Viscosity

Viscosity was determined in triplicate with the rotational viscometer J.P. Selecta ST-2020-L (Barcelona, Spain) with the S2 spindle at 40 rpm and the results were expressed in mPa.s. [33].

#### 2.3.3. Mineral Composition

The mineral composition was measured using the inductively coupled plasma mass spectrometry (ICP-MS) Shimadzu MS-2030 (Shimadzu, Kioto, Japan) using the conditions described by Muñoz-Bas et al. [11]. To analyze the mineral content, the samples were lyophilized (Freeze dryer Alpha 2–4, Martin Christ Gefriertrocknungsanlagen GmbH, Germany) and digested with nitric acid (67%) and hydrogen peroxide (33%) using a microwave system. The results were measured in triplicate and expressed as mg/100 g wet weight of kefir samples.

#### 2.3.4. Organic Acids and Sugars

Organic acids and sugars were extracted using the method described by Muñoz-Bas et al. [25]. After extraction was completed, 20 μL of each sample was injected into an HPLC (Hewlett-Packard 1100 series, Woldbronn, Germany) with a Supelco SupelcogelTM c-610H column (300 mm × 7.8 mm). The elution buffer used was orto-phosphoric acid in water (0.1% *v*/*v*) and organic acids were quantified by measuring the absorbance at 210 nm with a diode array detector (DAD G-1315 A, Agilent, Santa Clara, CA, USA), while sugars were evaluated using a refractive index detector (RID G1362A, Agilent, Santa Clara, CA, USA). The peaks were identified by comparing their retention times with those of the standards (organic acids, monosaccharides and oligosaccharides from Supelco, Sigma-Aldrich, St. Louis, MO, USA) and quantified using the regression formula derived from the standards.

#### 2.3.5. Microbiology

The microbiological quality (total aerobic count, LAB, Enterobacteriaceae and molds and yeast) of kefir with date coproducts was evaluated following the procedure described by Muñoz-Tebar et al. [34]. A total of 10 g of each kefir sample was homogenized with 90 mL of sterile peptone water 0.1% (*w*/*vol*) in a masticator for 60 s. Decimal dilutions were then made using the same medium and 0.1 mL was manually seeded, in duplicate, on MRS agar for *Lactobacillus* spp. and on M17 agar for *Streptococcus* spp. Petri dishes were incubated at 37 °C for 48 h in the case of *Streptococcus* spp., and at 37 °C for 48 h in an anaerobic chamber with an Anaerocult A (Merck, Darmstadt, Germany) for *Lactobacillus* spp. Molds and yeasts and Enterobacteriaceae were also counted using the same dilutions, and 1 mL of these dilutions was seeded, in duplicate, in Petrifilm plates (3 M, Madrid, Spain) for molds and yeasts and for Enterobacteriaceae. Petrifilm dishes were incubated at 37 °C for 24 h for the Enterobacteriaceae, and at 25 °C for 120 h for molds and yeasts. Plates with 30–300 colony-forming units (CFU) were manually counted and the results were expressed as log CFU/g of kefir.

#### 2.3.6. Sensory Analysis

To evaluate the acceptance of kefir with date paste, a consumer study was carried out at the Polytechnic School of Orihuela of Miguel Hernández University (UMH) with fifty consumers (55% female, 45% male, aged from 20 to 65). Prior to commencing the analyses, all participants were briefed on the distinct features of the product they would be tasting and the nature of the analysis itself. They also provided their written informed consent. This study received approval from the Responsible Research Office at Miguel Hernández University (OIR-Reg. 211128200759, Ref. PRL.DTA.JPA.05.21, UMH, Elche, Alicante, Spain). The study was carried out in a special room for sensory analysis studies which complied with international standards [35]. Participants of the study were seated in individual booths under TL 5 fluorescent lighting (Philips-Iberica, Madrid, Spain) with an intensity of approximately 350 lx. The kefir samples were served in small transparent plastic cups, each with a label with three different digits and in a random order. Consumers evaluated the following seven attributes (color, odor, flavor, sweetness, acidity, viscosity and overall acceptability). A discrete nine-point hedonic scale ranging from “dislike extremely” (1) to “like extremely” (9) was used [36].

### 2.4. Statistical Analysis

Statistics were measured using SPSS (IBM SPSS Statistics version 26). A one-way ANOVA (using a 95% confidence level) was performed on the results obtained to determine any significant differences between the kefir control and the samples containing date paste (3% and 6%) and cheese whey (25% and 50% milk substitution). When there was a significant difference, a Tukey test was performed to check for differences among the kefir formulations.

## 3. Results and Discussion

### 3.1. Proximate Composition

Table 1 shows the proximate composition of kefir influenced by the incorporation of date paste and goat cheese whey, where it can be seen that the values observed in all samples were within the proximate composition ranges reported in the literature for goat milk kefir [37,38,39]. An increase in moisture and ash content was observed in all samples (both those containing whey and date paste) compared to the control kefir, with the highest values found in the kefir made with 50% cheese whey as a milk substitute. The significant increase in moisture content in the WH50 sample may be attributed to the whey itself, as whey is primarily water (>90%; [40,41]). Similarly, fat and protein content significantly decreased (*p* < 0.05) in the samples with date paste and cheese whey compared to the control, dropping from 4.87% to 2.68% fats, and from 3.73% to 1.63% proteins in the WH50 kefir. Finally, for total solids, a significant decrease (*p* < 0.05) was observed in kefir containing date paste (DP3 and DP6, *p* > 0.05) and an even greater decrease in kefir containing whey (WH25 and WH50, *p* < 0.05). Overall, the differences found among the formulations are likely related to the proximate composition of the date paste (low protein and fat content) and cheese whey (low fat and protein content and high water content) reported in Section 2.1.

In comparison to similar studies, Erzhad et al. [42] also reported lower protein and higher ash values when they incorporated red fruit extracts in probiotic goat milk beverages, while Tawfeck et al. [43] observed lower fat levels in fermented goat milk drinks enriched with date palm compared to control kefir.

### 3.2. Mineral Profile

The mineral content of kefir formulated with date paste and cheese whey is presented in Table 2, showing significant changes in all minerals analyzed (*p* < 0.05). The minerals found in greater amounts were sodium (95.06–423.58 mg/100 g), potassium (95.45–131.37 mg/100 g), calcium (64.04–88.68 mg/100 g), phosphorus (39.92–64.61 mg/100 g) and magnesium (8.04–11.43 mg/100 g), while iron, copper, manganese and zinc were found in small amounts (<1 mg/100 g). Notably, the addition of date paste resulted in a significant increase in Ca, P and Zn levels, whereas samples containing cheese whey showed the lowest values for these minerals. Similarly, K, Mg, Cu and Mn levels were higher in kefir enriched with date paste, while the substitution of milk with cheese whey had no significant effect on them as their values were similar to the control (*p* > 0.05). The observed increases in the Ca, K and Mg content in kefir enriched with date paste are primarily attributed to the mineral content of the date paste itself, which contains substantial amounts of these macroelements (K: 359.36 mg/100 g, Ca: 272 mg/100 g, Mg: 165.05 mg/100 g) [25]. Since the most valuable mineral in kefir is calcium, the reformulation of kefir with up to 25% date paste or even with cheese whey did not cause a decrease in its content. On the other hand, it was observed that the iron content decreased when incorporating 50% date paste and cheese whey, while the values remained the same in the control kefir and the one containing 25% whey. However, these differences were practically insignificant, as Fe concentrations across all samples were consistently low (0.04–0.05 mg/100 g). The most pronounced effect was found in the sodium content, which was significantly increased in the kefir formulated with cheese whey, obtaining values almost 5 times higher (WH50) compared to the control (95.06 vs. 423.58 mg/100 g). Sodium, part of the milk’s aqueous phase, is found in greater quantities in whey, also being influenced by the salt used in cheese processing which could explain the highest content of sodium being present in kefir formulated with cheese whey compared to the control or those containing date paste. In contrast, copper, iron and zinc, which are more strongly associated with caseins in ruminant milk, are present in lower concentrations in whey [44].

In a similar study, Tawfek et al. [43] developed fermented goat milk beverages with date palm, reporting comparable results in mineral content with significant increases in potassium, magnesium, iron and zinc content. So far, the available literature on minerals in fermented goat milk products has been limited since most studies have mainly focused on the mineral composition of the milk itself. Therefore, the findings of the present study are particularly valuable, offering new insights that can support the development of innovative fermented goat milk products, specifically those enriched with date paste and cheese industry by-products such as whey.

### 3.3. Organic Acids and Sugars

The analysis of organic acids and sugars in goat kefir (Table 3) revealed that lactic acid was the only organic acid consistently detected across all formulations. The lowest levels (*p* > 0.05) were observed in samples containing whey, primarily due to the lactic acid content typically found in cheese whey, as noted by Borba et al. [45]. This reduced lactic acid content in whey results from the cheese-making process, which includes milk pasteurization and the addition of rennet to coagulate casein and form curds, without significant lactobacilli activity to convert lactose into lactic acid [45].

In formulations enriched with date paste, an increase in lactic acid was noted compared to the control (from 5.55 to 6.42 mg/g). This increase in lactic acid content was mainly due to the fact that LAB such as *Lactobacillus* spp. use glucose as a substrate during fermentation to produce lactic acid [46].

Regarding sugars, lactose was the predominant sugar found in all goat kefir formulations, while fructose was only detected in samples containing date paste. This was because fructose is one of the primary sugars in date paste, as previously reported (137.72 mg/g) [25]. On the other hand, the highest lactose levels were observed in samples enriched with cheese whey, primarily due to the naturally high lactose content in whey [44,45]. However, the addition of date paste did not affect the lactose content of kefir, with values remaining comparable to the control.

### 3.4. Physicochemical Properties

Considering that kefir is defined as “a refreshing fermented dairy product with flowing consistency, and uniform and bright appearance” [47], it seems logical that the most influential properties on its quality were those relating to the lactic fermentation process (acidity and pH), flow properties (viscosity) and color properties (L*, a* and b* coordinates and C* and H*). Table 4 shows the results of the physicochemical properties of kefir samples, demonstrating that all of them were significantly affected by the reformulation of kefir.

Titratable acidity (TA) and pH play a key role in the texture and flavor of fermented dairy products like kefir. In particular, the acidity levels in kefir are important, as both insufficient and excessive acidity can mask its characteristic buttery flavor and alter the product’s structure [48]. Although there is no direct correlation between these two parameters, a general trend exists since pH decreases as TA increases [49]. Furthermore, both parameters are indicators of good quality, not only during the production of fermented dairy products but also of the final product. The pH and TA values (^0^Dornic) of the different kefir samples are shown in Table 4, showing that the addition of DP slightly decreased the pH values of kefir in a concentration-dependent manner (*p* < 0.05). Similar behavior was observed as a function of the cheese whey concentration, reaching pH values similar to those of the DP-added kefir. The decrease in pH values in kefir due to the addition of fruit extracts (such as pomegranate peel, mango peel, red prickly pear, lemon fiber and apple fiber, among others) has been previously reported [16,50,51]. This reduction was attributed to the phytochemicals and organic acids present in fruits, which can contribute to the acidic pH in kefir. In addition, fruit extracts can serve as a source of natural sugars and polysaccharides, which can be converted into glucose and eventually transformed into lactic acid by microorganisms [51]. Nevertheless, the pH values of all kefir samples were in the pH range normally reported for kefir samples (3.5–4.5; [52,53]). Titratable acidity showed a different trend, though. DP-added kefir (DP3 and DP6) showed similar TA values (*p* > 0.05) compared to the control kefir. However, the addition of whey led to a concentration-dependent decrease in TA values (*p* < 0.05), with the largest difference observed between the control and WCH50. In any case, as the values of TA normally reported for kefir samples vary between 0.50 and 1.50 g/100 mL [52,53], it could be said that all kefir samples showed values in this range. It is also important to note that none of the samples had pH values below 4.0, as such low pH levels are considered a detrimental level for probiotics [16].

The consistency (viscosity) of kefir is influenced by several factors, with the type of milk and the fermentation process (grain vs. lyophilized culture, and incubation temperatures) being the most important. Studies have shown that kefir made from goat and sheep milk has considerably lower viscosity compared to that made from cow milk, and that the use of kefir grains at high temperatures results in higher viscosity values [47,54,55]. The addition of DP did not modify (*p* > 0.05) the viscosity in the kefir samples at any of the added concentrations (3% and 6%). In other words, DP could be used as an ingredient in fortified kefir (up to 6%) without altering its flow properties. It has been reported that the addition of DP in other dairy products (yogurt and cheese) did not cause significant changes in their texture because DP was perfectly integrated within the milk matrix [25,34]. However, the substitution of goat milk by cheese whey significantly reduced the viscosity values, with this decrease being higher at higher substitution percentages (*p* < 0.05). These results were expected, knowing that cheese whey has a lower viscosity than goat milk.

Color plays a relevant role in the production and marketing of foods, particularly in fermented dairy products, which are typically known for their characteristic white color. It can significantly influence consumer acceptance and purchase decisions. In kefir, the color can vary due to several factors, highlighting the type of milk, the specific fermentation process and any added ingredients or additives (flavorings, colorants, etc.). The manner and extent of these color variations could even hinder the development and market launch of a new dairy product. All the reformulated kefir samples showed L* values lower than the control kefir (*p* < 0.05), with the WH50 showing the lowest L*. The lightness value in the control kefir was in line with the L* values of commercial kefir [38]. The decrease in L* values in kefir due to the addition of fruit extracts (lemon fiber, apple fiber and red prickly pear) has also been reported by other authors [16,56]. Regarding the a* and b* values, a significant increase (*p* < 0.05) was observed in DP-added kefir samples (DP3 and DP6), without any differences between the concentrations compared to the control kefir. These samples displayed the highest a* and b* values. The addition of whey only increased a* and b* values when it was added at 25% (*p* < 0.05), with WH50 showing similar a* and b* values to control kefir. The changes in redness and yellowness in kefir due to DP addition was related to the corresponding values of the date paste (a*: 2.5 and b*: 4.9; [25]) due to its orange–yellow–red pigment content (carotenes and anthocyanins) and also due to some browning compounds generated during Maillard reactions [25,57]. The color saturation behavior in kefir due to its reformulation followed the same pattern as the b* coordinate. The addition of DP increased C* values, while the use of cheese whey at 50% resulted in saturation values similar to those of the control. Hue tone decreased in reformulated kefir (except in WH50 which showed a hue similar to control; *p* < 0.05). Hue is the dimension that most people associate with an object’s color; therefore, the reformulation of kefir caused a shift in hue from a lemon-yellow hue (control) to yellow–orangish (DP3 and DP6) and lemonish-yellow (WH25) [38,58]. The whiteness index decreased (*p* < 0.05) with the addition of date coproducts. In DP-added kefir, the WI did not show differences between the concentrations of DP added (*p* > 0.05). Kefir with cheese whey at the highest concentration (WH50) showed the lowest WI (*p* < 0.05). However, the WI values obtained for all reformulated kefir samples were in the range of the values reported for commercial kefir samples [38].

### 3.5. Microbiology

The microbiological analysis results for goat milk kefir enriched with date paste and cheese whey added with date are presented in Table 5. These results prove that the incorporation of both date paste and cheese whey did not impair the growth of kefir starter LAB and the values met the recommended minimum of 10^6^ CFU/mL for live probiotic bacteria in probiotic food products [59]. The presence of Enterobacteriaceae was not detected in any kefir formulation, which confirmed that the process was carried out under optimal hygienic conditions and that the raw materials used (milk, date paste and cheese whey) were free from contamination by this type of bacteria.

For the total mesophilic aerobic counts, kefir samples enriched with date paste exhibited the highest values (*p* < 0.05) compared to the control, followed by samples containing cheese whey. In contrast, for Streptococcus sp., the control kefir showed the highest counts (8.27 log CFU/mL), followed by kefir enriched with date paste (8.23 and 8.25 log CFU/mL). The lowest counts were observed in samples where part of the milk (25% and 50%) was replaced by cheese whey (8.21 and 8.18 log CFU/mL, respectively). Regarding Lactobacillus sp. counts, a significant increase (*p* < 0.05) was observed with the incorporation of date paste and cheese whey (enriched with 8% date paste), showing values ranging from 8.21 to 8.47 log CFU/mL. This significant increase in date-enriched formulations suggests that their probiotic potential could be related to the previous prebiotic activity of date coproducts, as previously demonstrated in the study carried out by Muñoz-Bas et al. [28]. Additionally, the improvement of microbial growth in samples fortified with date paste might be linked to its mineral and phenolic compound contents, which could serve as prebiotic substrates in functional fermented products, as well as its carbohydrate content, which may stimulate the LAB [60]. However, the interaction between bacteria and phenolic compounds is complex and can be influenced by factors such as the type and concentration of the substrate and the specific bacterial strain involved [61,62]. Finally, molds and yeasts were only detected in the samples formulated with cheese whey, but their growth was so low (<3 log cfu/mL) that they did not represent any risk for consumption. Considering that the yeast *Kluyveromyces marxianus* was in the starter used in the kefir elaboration, its absence in the kefir samples could be due to multiple factors such as competition with other microorganisms, changes in nutrient availability or the use of the culture medium for molds and yeasts instead of a selective one for yeasts.

The results obtained in the present work are in line with the study conducted by Tawfek et al. [43] on date-enriched fermented goat milk beverages, which also showed higher counts of *Lactobacillus* spp. and *Sreptococcus* spp. compared to the control sample.

### 3.6. Sensory Analysis

**Figure 1:**
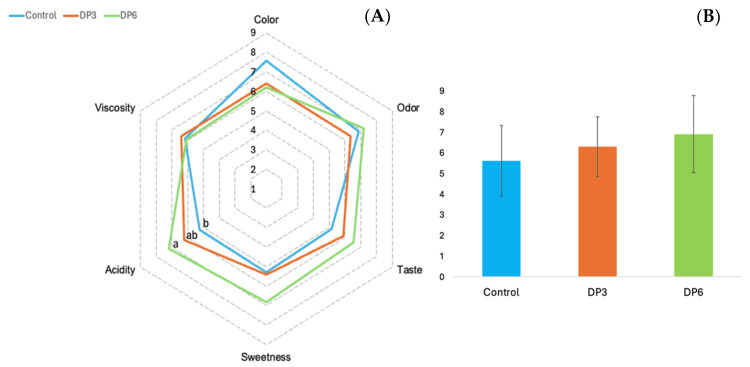
Sensory evaluation (**A**) and overall acceptance (**B**) of fortified kefir. Control: kefir without date paste added; DP3: kefir with 3% date paste added; DP6: kefir with 6% date paste added. Data are presented as mean ± SD. Different letters indicate statistically significant differences as determined by Tukey’s HSD post hoc test (*p* < 0.05).

The kefir samples containing the cheese whey as a milk substitute were excluded from the sensory analysis. The whey caused phase separation in the kefir, resulting in the loss of its typical appearance with the result being unsuitable for sensory analysis. The consumer panelists evaluated six attributes in each of the samples (Figure 1).

Significant differences (*p* < 0.05) were only found in acidity. The control sample had the lowest acidity score while the highest score was given to the kefir formulated with 6% DP. No significant differences were found for the rest of the attributes (color, odor, flavor, sweetness and viscosity). However, the kefir with 6% DP received the highest scores in several of these attributes, including odor, flavor, sweetness and acidity. Finally, in terms of overall acceptability, the sample that consumers liked the most was the one formulated with 6% date paste.

## 4. Conclusions

Fermented dairy products play an important role in supporting a healthy lifestyle and can offer numerous health advantages. Current trends emphasize the use of probiotics and plant extracts, mainly from agro-industry coproducts, to improve their sustainability. This study demonstrates that the use of date coproducts (date paste and cheese whey from the elaboration of fresh cheese with date paste) for the fortification of goat milk kefir is technologically feasible (although the use of whey would need further technological improvement), contributing to the growth of the dairy industry and expanding the range of available options in the market. The addition of both ingredients promoted the growth of probiotic microorganisms. The addition of date paste also improved the mineral profile of goat milk kefir (with the exception of sodium content). Date paste could be used as an ingredient in fortified kefir (up to 6%) without altering its flow properties, because it could be perfectly integrated within the milk matrix. The use of date–cheese whey as a goat milk substitution (>25%) decreased the typical kefir viscosity, inducing an excessive phase separation negatively valued by consumers. Consumers preferred the kefir with 6% date paste, mainly due to its higher scores for odor, flavor, sweetness and acidity. For the use of cheese whey, it should be necessary to change its processing to avoid the phase separation which was responsible for the loss of the typical kefir appearance and texture, making it less acceptable to consumers.

## Figures and Tables

**Table 1:** Nutritional composition (g/100 g) of goat kefir fortified with date coproducts (mean ± sd).

Sample	Protein	Fat	Ash	Moisture	Total Solids
Control	3.73 ± 0.12 ^a^	4.87 ± 0.04 ^a^	0.72 ± 0.01 ^c^	85.42 ± 0.32 ^c^	4.85 ± 0.21 ^a^
DP3	2.39 ± 0.01 ^b^	3.04 ± 0.00 ^b^	0.76 ± 0.02 ^bc^	86.56 ± 0.83 ^bc^	4.36 ± 0.06 ^b^
DP6	2.46 ± 0.03 ^b^	3.22 ± 0.80 ^b^	0.85 ± 0.05 ^b^	86.73 ± 0.19 ^ab^	4.40 ± 0.07 ^b^
WH25	2.11 ± 0.07 ^c^	3.17 ± 0.04 ^b^	0.82 ± 0.02 ^bc^	86.08 ± 0.59 ^bc^	3.81 ± 0.01 ^c^
WH50	1.63 ± 0.01 ^d^	2.68 ± 0.17 ^c^	0.98 ± 0.04 ^a^	87.96 ± 0.62 ^a^	3.16 ± 0.05 ^d^
*p*-value	0.000	0.000	0.000	0.001	0.000

^a–d^ Different letters between rows mean significant differences (*p* < 0.05). DP3: kefir with 3% date paste; DP6: kefir with 6% date paste; WH25: kefir with 25% of milk substituted by date–cheese whey; WH50: kefir with 50% of milk substituted by date–cheese whey.

**Table 2:** Mineral profile (mg/100 g) of kefir fortified with date coproducts (mean ± sd).

Sample	Control	DP3	DP6	WH25	WH50	*p*-Value
Ca	82.09 ± 1.70 ^b^	88.16 ± 0.86 ^a^	88.68 ±1.29 ^a^	80.32 ± 0.28 ^b^	64.04 ± 1.28 ^c^	0.000
Cu	0.01 ± 0.00 ^b^	0.01 ± 0.00 ^ab^	0.02 ± 0.00 ^a^	0.01 ± 0.00 ^b^	0.01 ± 0.00 ^b^	0.000
Fe	0.05 ± 0.00 ^a^	0.04 ± 0.00 ^b^	0.04 ± 0.00 ^b^	0.05 ± 0.00 ^a^	0.04 ± 0.00 ^b^	0.000
K	96.64 ± 1.84 ^b^	131.25 ± 0.45 ^a^	131.37 ± 0.75 ^a^	97.74 ± 1.81 ^b^	95.45 ± 2.48 ^b^	0.000
Mg	8.47 ± 0.11 ^c^	10.39 ± 0.10 ^b^	11.43 ± 0.08 ^a^	8.27 ± 0.45 ^c^	8.04 ± 0.19 ^c^	0.000
Mn	0.01 ± 0.00 ^b^	0.01 ± 0.00 ^b^	0.02 ± 0.00 ^a^	0.01 ± 0.00 ^b^	0.01 ± 0.00 ^b^	0.000
Na	95.06 ± 5.25 ^d^	104.83 ± 1.57 ^c^	111.23 ± 3.37 ^c^	376.86 ± 0.84 ^b^	423.58 ± 0.66 ^a^	0.000
P	54.33 ± 0.01 ^c^	58.06 ± 0.77 ^b^	64.61 ± 0.83 ^a^	55.25 ± 0.68 ^c^	39.92 ± 0.63 ^d^	0.000
Zn	0.23 ± 0.01 ^c^	0.30 ± 0.001 ^b^	0.36 ± 0.02 ^a^	0.22 ± 0.01 ^c^	0.17 ± 0.01 ^d^	0.000

^a–d^ Different letters between columns mean significant differences (*p* < 0.05). DP3: kefir with 3% date paste; DP6: kefir with 6% date paste; WH25: kefir with 25% milk substituted by date–cheese whey; WH50: kefir with 50% milk substituted by date–cheese whey.

**Table 3:** Sugar and organic acid contents (mg/g) of kefir fortified with date coproducts (mean ± sd).

Sample	Lactic Acid	Fructose	Lactose
Control	5.55 ± 0.16 ^b^	ND	27.06 ± 0.89 ^c^
DP3	6.28 ± 0.07 ^a^	4.37 ± 0.02 ^b^	27.25 ± 0.72 ^c^
DP6	6.42 ± 0.09 ^a^	5.68 ± 0.02 ^a^	26.93 ± 0.19 ^c^
WH25	3.21 ± 0.18 ^c^	ND	34.22 ± 0.04 ^b^
WH50	2.89 ± 0.17 ^d^	ND	36.38 ± 0.03 ^a^
*p*-value	0.001	0.000	0.000

^a–d^ Different letters between rows mean significant differences (*p* < 0.05). DP3: kefir with 3% date paste; DP6: kefir with 6% date paste; WH25: kefir with 25% milk substituted by date–cheese whey; WH50: kefir with 50% milk substituted by date–cheese whey.

**Table 4:** Physicochemical properties of kefir fortified with date coproducts (mean ± sd).

Sample	Control	DP3	DP6	WH25	WH50	*p*-Value
pH	4.32 ± 0.02 ^a^	4.24 ± 0.01 ^b^	4.21 ± 0.01 ^c^	4.26 ± 0.01 ^b^	4.20 ± 0.01 ^c^	0.000
Acidicity (^0^D)	94.00 ± 2.83 ^a^	101.00 ± 1.41 ^a^	96.50 ± 2.12 ^a^	64.00 ± 1.41 ^b^	66.00 ± 1.41 ^c^	0.000
Viscosity (mPa.s)	1125.80 ± 60.37 ^a^	1193.10 ± 1.18 ^a^	1141.10 ± 6.20 ^a^	451.60 ± 12.82 ^b^	113.27 ± 4.02 ^c^	0.000
L* (D65)	81.186 ± 0.74 ^a^	73.83 ± 0.71 ^b^	77.36 ± 0.57 ^b^	78.48 ± 0.57 ^c^	61.12 ± 0.39 ^d^	0.000
a* (D65)	−0.74 ± 0.09 ^c^	0.77 ± 0.18 ^a^	0.67 ± 0.08 ^a^	−0.23 ± 0.03 ^b^	−0.62 ± 0.05 ^c^	0.000
b* (D65)	5.14 ± 0.18 ^b^	8.67 ± 0.54 ^a^	9.18 ± 0.53 ^a^	8.39 ± 0.19 ^a^	4.55 ± 0.02 ^b^	0.000
C* (D65)	5.19 ± 0.17 ^b^	8.70 ± 0.54 ^a^	9.21 ± 0.53 ^a^	8.39 ± 0.19 ^a^	4.59 ± 0.03 ^b^	0.000
H* (D65)	98.24 ± 1.16 ^a^	84.92 ± 1.14 ^c^	85.81 ± 0.19 ^c^	91.59 ± 0.14 ^b^	97.73 ± 0.60 ^a^	0.000
WI	80.48 ± 0.72 ^a^	75.73 ± 0.41 ^b^	76.60 ± 0.72 ^b^	72.51 ± 0.62 ^c^	60.84 ± 0.38 ^d^	<0.001

^a–d^ Different letters between columns mean significant differences (*p* < 0.05). WI (whiteness index); DP3: kefir with 3% date paste; DP6: kefir with 6% date paste; WH25: kefir with 25% milk substituted by date–cheese whey; WH50: kefir with 50% milk substituted by date–cheese whey.

**Table 5:** Microbiological counts (log CFU/ g) of kefir fortified with date coproducts (mean ± sd).

Sample	Total Aerobic Count	*Lactobacillus* sp.	*Streptococcus* sp.	Enterobacteriaceae	Molds	Yeasts
Control	8.36 ± 0.03 ^bc^	8.21 ± 0.01 ^d^	8.27 ± 0.01 ^a^	ND	ND	ND
DP3	8.41 ± 0.01 ^ab^	8.47 ± 0.01 ^a^	8.25 ± 0.01 ^ab^	ND	ND	ND
DP6	8.45 ± 0.00 ^a^	8.44 ± 0.01 ^a^	8.23 ± 0.02 ^ab^	ND	ND	ND
WH25	8.29 ± 0.02 ^c^	8.32 ± 0.01 ^c^	8.21 ± 0.01 ^bc^	ND	1.54 ± 0.09 ^b^	1.98 ± 0.03 ^b^
WH50	8.33 ± 0.02 ^bc^	8.37 ± 0.01 ^b^	8.18 ± 0.00 ^c^	ND	1.81 ± 0.05 ^a^	2.83 ± 0.02 ^a^
*p*-value	0.003	0.000	0.004	ND	0.000	0.000

^a–d^ Different letters between rows mean significant differences (*p* < 0.05). ND: not detected. DP3: kefir with 3% date paste; DP6: kefir with 6% date paste; WH25: kefir with 25% milk substituted by date–cheese whey; WH50: kefir with 50% milk substituted by date–cheese whey.

## Data Availability

The original contributions presented in this study are included in the article. Further inquiries can be directed to the corresponding author.
